# Extent and correlates of self-reported exposure to tobacco advertising, promotion and sponsorship in smokers: Findings from the EUREST-PLUS ITC Europe Surveys

**DOI:** 10.18332/tid/94828

**Published:** 2019-01-17

**Authors:** Sarah Kahnert, Tibor Demjén, Yannis Tountas, Antigona C. Trofor, Krzysztof Przewoźniak, Witold A. Zatoński, Esteve Fernández, Ann McNeill, Marc Willemsen, Christina N. Kyriakos, Geoffrey T. Fong, Constantine I. Vardavas, Ute Mons, on behalf of the EUREST-PLUS consortium

**Affiliations:** 1 Cancer Prevention Unit and WHO Collaborating Centre for Tobacco Control, German Cancer Research Center (DKFZ), Heidelberg, Germany; 2 Medical Faculty, Heidelberg University, Heidelberg, Germany; 3 Smoking or Health Hungarian Foundation (SHHF), Budapest, Hungary; 4 National and Kapodistrian University of Athens (UoA), Athens, Greece; 5 University of Medicine and Pharmacy “Grigore T. Popa” Iasi, Iasi, Romania; 6 Aer Pur Romania, Bucharest, Romania; 7 Health Promotion Foundation (HPF), Warsaw, Poland; 8 Oncology Center, Maria Sklodowska-Curie Institute, Warsaw, Poland; 9 European Observatory of Health Inequalities, President Stanisław Wojciechowski State University of Applied Sciences, Kalisz, Poland; 10 Tobacco Control Unit, Catalan Institute of Oncology (ICO), Catalonia, Spain; 11 Cancer Control and Prevention Group, Bellvitge Biomedical Research Institute (IDIBELL), Catalonia, Spain; 12 King’s College London (KCL), London, United Kingdom; 13 Maastricht University, Maastricht, the Netherlands; 14 Netherlands Expertise Center for Tobacco Control (Trimbos Institute), Utrecht, the Netherlands; 15 European Network for Smoking and Tobacco Prevention (ENSP), Brussels, Belgium; 16 University of Crete (UoC), Heraklion, Greece; 17 Department of Psychology, University of Waterloo (UW), Waterloo, Canada; 18 School of Public Health and Health Systems, University of Waterloo (UW), Waterloo, Canada; 19 Ontario Institute for Cancer Research, Toronto, Canada

**Keywords:** tobacco marketing, tobacco advertising, tobacco marketing restrictions, regulatory science, Europe

## Abstract

**INTRODUCTION:**

Tobacco advertising, promotion and sponsorship (TAPS) are known to promote tobacco consumption and to discourage smoking cessation. Consequently, comprehensive TAPS bans are effective measures to reduce smoking. The objective of this study was to investigate to what extent smokers are exposed to TAPS in general, and in various media and localities, in different European countries.

**METHODS:**

A cross-sectional analysis of national representative samples of adult smokers in 2016 from Germany, Greece, Hungary, Poland, Romania, and Spain (EUREST-PLUS Project, n=6011), as well as England (n=3503) and the Netherlands (n=1213) (ITC Europe Surveys) was conducted. Prevalence of self-reported TAPS exposure is reported by country, and socioeconomic correlates were investigated using logistic regression models.

**RESULTS:**

Self-reported exposure to TAPS varied widely among the countries, from 15.4 % in Hungary to 69.2 % in the Netherlands. In most countries, tobacco advertising was most commonly seen at the point of sale, and rarely noticed in mass media. The multivariate analysis revealed some variation in exposure to TAPS by sociodemographic factors. Age showed the greatest consistency across countries with younger smokers (18–24 years) being more likely to notice TAPS than older smokers.

**CONCLUSIONS:**

TAPS exposure tended to be higher in countries with less restrictive regulation but was also reported in countries with more comprehensive bans, although at lower levels. The findings indicate the need for a comprehensive ban on TAPS to avoid a shift of marketing efforts to less regulated channels, and for stronger enforcement of existing bans.

## INTRODUCTION

Tobacco advertising, promotion and sponsorship (TAPS) are used by tobacco companies to create positive product and company imagery and associations, with the aim to increase sales^1^. The tobacco industry utilizes a wide spectrum of legally available marketing measures; including direct marketing, such as advertising in mass media (TV, radio, print), on the internet, through outdoor advertising, or at the point of sale; and indirect marketing, such as promotional activities and sponsorship^2^.

Although the tobacco industry claims to target only adult smokers, it is well-established that tobacco marketing promotes tobacco use among adolescents^3-5^. It has also been shown that tobacco advertising encourages smokers to increase consumption^6^ and interferes with smoking cessation^7-9^.

Comprehensive bans on TAPS are known to be effective measures to reduce smoking prevalence^10^, while partial marketing restrictions have little or no effect because marketing efforts are shifted to less regulated channels^1,6^. Thus, the World Health Organization Framework Convention on Tobacco Control (WHO FCTC) calls for comprehensive bans on all types of direct and indirect marketing, including cross-border TAPS (WHO FCTC, Article 13)^11^. However, more than ten years after the WHO FCTC came into force, and despite efforts to harmonize advertising regulations across member states of the European Union (EU), there is still some heterogeneity regarding TAPS legislation in Europe^12^.

In 2003, several forms of advertising and sponsorship were prohibited at EU level by the Tobacco Advertising Directive (2003/33/EC)^13^. The ban covers advertising in printed media and on the internet, radio advertising and sponsorship, sponsorship of events or activities involving or taking place in several Member States or otherwise having cross-border effects (e.g. *Formula 1* races), as well as any free distribution of tobacco products at such events. However, other forms of direct marketing, e.g. outdoor and point of sale advertising, and indirect marketing, e.g. sponsorship of events without cross-border effects, are regulated at national or local level.

While some European countries such as Hungary, Poland and the UK are quite progressive with regards to TAPS bans, others such as Germany or Greece still lack restrictions on several types of advertising, likely leading to differences across EU countries in TAPS exposure. Thus, the aim of this paper was to study EU cross-country differences in self-reported exposure to TAPS in various media (TV, radio, print, online, billboards) and localities (bars/pubs, points of sale, events). To gain insight into differential tobacco promotion exposure of vulnerable groups, socioeconomic and sociodemographic correlates of exposure were examined overall and within countries. Furthermore, awareness of advertising and information on the dangers of smoking or that encourages cessation, as well as endorsement of tobacco advertising bans at points of sale, were explored.

## METHODS

### Study design

This study was conducted within the context of the European Commission Horizon 2020 funded study entitled **European Regulatory Science on Tobacco: Policy implementation to reduce lung diseases (EUREST-PLUS-HCO-06-2015).** The EURESTPLUS Project^14,15^, which involves the creation of a cohort of adult smokers in six EU Member States (Germany, Greece, Hungary, Poland, Romania, Spain; n=6011) aims to assess the implementation of the Tobacco Products Directive (2014/40/EU)^16^ and the WHO FCTC at the European level. The conceptual model of EUREST-PLUS is based on the theory-driven framework of the International Tobacco Control Policy Evaluation Project (ITC), which hypothesizes the pathways of tobacco control polices on tobacco use behaviours^17^. Data from the first wave of this ITC 6 European Country (ITC 6E) Survey were used for this study. Because all ITC surveys are based on the same methodology and use standardized survey questionnaires^18^, it was possible to additionally use cross-sectional data from the first wave of the ITC Four Country Tobacco and E-Cigarette (ITC 4CE1) Survey in England, and from the ITC Netherlands (ITC NL) Survey.

### Data collection

The ITC 6E sample, collected between 18 June and 12 September 2016, comprised 6011 nationally representative smokers (i.e. adult cigarette smokers) aged 18 years or older (about 1000 in each of the project six countries). The geographic strata were regions according to the Classification of Territorial Units for Statistics (NUTS) crossed with degree of urbanization (urban, intermediate, rural). Approximately 100 area clusters were sampled in each country, with the aim of obtaining 10 smokers per cluster. Clusters were allocated to strata proportionally to an 18 years and older population size. Within each cluster, household addresses were sampled using a random walk design. One randomly selected male smoker and one randomly selected female smoker were chosen for interview from a sampled household, where possible. Screening of households continued until the required number of smokers from the cluster had been interviewed. All interviews were conducted face-to-face by interviewers using tablets (Computer Assisted Personal Interview, CAPI). For further details, see the ITC 6E Wave 1 Technical Report^19^.

Data for Wave 1 of ITC 4CE1 Survey were collected in England between 7 July and 16 November 2016. The sample comprised the following cohorts: 1) recontacted smokers and quitters living in England who participated in Wave 10 of the earlier ITC 4C Project in the UK, regardless of e-cigarette use; 2) newly recruited current smokers and recent quitters (quit smoking in the past 24 months) from a commercial online panel, regardless of e-cigarette use; and 3) newly recruited current e-cigarette users (use at least weekly) from a commercial online panel. In sampling, quotas obtained from national survey data for region crossed with male/female were applied to cohorts 2) and 3). For further details on methods and data collection, see the ITC 4CE Wave 1 Technical Report^20^. Only data from current adult cigarette smokers were used for this study.

Data for Wave 10 of the ITC NL Survey were collected in the Netherlands between 15 November and 31 December 2016. Respondents were 1696 adults aged 15 years or older recruited as cigarette smokers, who were members of a commercial online panel. The nationally representative sample included 1318 subjects who had also responded in Wave 9, and 378 new respondents recruited to replenish dropouts. Again, only current adult smokers were included. For further details on methods of data collection, see the ITC NL Wave 10 Technical Report^21^.

### Ethics procedures

The study was approved by the Research Ethics Board of the University of Waterloo, Ontario, Canada and by local ethics boards within the study countries. Participation in the study was contingent on provision of individual informed consent, which was obtained either in written or verbal form according to local ethical requirements. The EUREST-PLUS Project is registered in Clinicaltrials.gov with trial registration number NCT02773836.

### Measures

The questionnaires included relevant sociodemographic variables, such as sex, age, marital status, education, and degree of urbanization. Age was categorized into four age groups (18–24, 25– 39, 40–54, 55 years and older). Marital status was classified into two groups (not married, widowed, divorced or separated; and not married but living together, married or registered partners). In each country, education was reclassified to match International Standard Classification of Education (ISCED) coding, which was, in turn, categorized into low (pre-Primary, Primary, lower Secondary), moderate (upper Secondary, post-Secondary non-Tertiary, short-cycle Tertiary), and high (Bachelor or equivalent, Master or equivalent, Doctoral or equivalent). The degree of urbanization comprised the three categories rural, intermediate and urban.

The number of cigarettes smoked per day (CPD) and self-reported time to the first cigarette of the day (TTF) were used to create the Heaviness of Smoking Index (HSI)^22^. CPD was categorized into less than 10, 11–20, 21–30, and 31 and more cigarettes, while the categories of TTF were more than 60 minutes, 31–60 minutes, 6–30 minutes, and 5 minutes or less. The HSI was calculated by summing the value of the categorical CPD and categorical TTF, both having category values from 0 to 3, which translates to the HSI having values ranging from 0 to 6. If either value was missing or coded as a non-response, then HSI was also classified as missing or non-response. According to the index, value smokers were subsequently categorized into three HSI groups (low 0–1: moderate 2–4: high 5–6).

To gather information on self-reported exposure to TAPS, respondents were asked ‘Thinking about everything that happens around you, in the last 6 months how often have you noticed things that promote smoking?… It doesn’t have to be advertising — anything that promotes smoking’. Response options were ‘never’, ‘rarely’, ‘sometimes’, ‘often’, ‘very often’ and ‘don’t know’, which were categorized into ‘yes’ (‘rarely’, ‘sometimes’, ‘often’, ‘very often’), ‘no’ (‘never’) and ‘don’t know’. Respondents who answered this question affirmatively were asked the following questions, with response options ‘yes’, ‘no’ and ‘don’t know’, about whether they had noticed, in the last 6 months, things that promote smoking in various media and localities, such as: a) television, b) radio, c) newspapers or magazines, d) social media sites, like Facebook, Twitter, YouTube, Instagram or Snapchat, e) the internet, f) posters or billboards, g) bars or pubs, h) outside shops or stores that sell tobacco, i) inside shops or stores that sell tobacco, and j) fairs, markets, festivals, sporting events, or music concerts. While all places were prompted in ITC 6E Survey, a)–c), f) and j) were not captured in ITC 4CE1 Survey, and f)–j) were not captured in ITC NL Survey. Additionally, in ITC 4CE1 Survey, there was a single question regarding ‘websites or social media sites’. Therefore, d) and e) were combined to one variable for comparative analysis. Exposure to things that promote smoking varies across countries and thus, even though site-specific exposure to TAPS was only asked amongst those who had noticed things that promote smoking, site-specific prevalence of exposure to TAPS was calculated with the whole sample as the denominator to allow for a more straightforward interpretation and better comparability of exposure prevalence.

Furthermore, in all surveys, respondents were asked if they had seen in the last 30 days tobacco packages (ITC 6E and ITC 4CE1 Surveys: ‘cigarette or roll-your-own tobacco packages’; ITC NL Survey: ‘cigarette packages’) ‘being displayed inside shops or stores where people can buy tobacco products, including on shelves or on the counter’ (ITC NL Survey does not refer to shops and stores).

To measure awareness of anti-smoking campaigns, respondents of ITC 6E and ITC NL Surveys, were asked: ‘Now I would like you to think about advertising or information that talks about the dangers of smoking or encourages quitting. In the last 6 months, how often have you noticed such advertising or information?’.

Moreover, in ITC 6E and ITC NL Surveys, but not in ITC 4CE1 Survey, support of complete bans ‘on tobacco advertisements inside shops and stores’ and ‘on displays of cigarettes inside shops and stores’ was inquired with the response options ‘not at all’, ‘somewhat’ and ‘a lot’ , which were categorized into ‘yes’ (‘somewhat’, ‘a lot’) and ‘no’ (‘not at all’).

### Statistical analysis

Percentages of exposure to TAPS in various media (TV, radio, print, online, billboards) and localities (bars/pubs, points of sale, events) were reported for each country. Exposure to things that promote smoking was additionally reported by sex, age group, education, marital status, level of urbanization (except for England and the Netherlands, as it was not captured in the surveys), and Heaviness of Smoking Index, while associations were tested for statistical significance using logistic regression models. All analyses incorporated weights derived from the complex sampling design. All statistical tests were two-sided, with an alpha level of 0.05. SAS v9.4 was used throughout.

## RESULTS

Table 1 shows the distribution of sociodemographic and socioeconomic characteristics, smoking status, and HSI, by country. In most countries, the majority of participants were male, middle aged, of low or moderate educational level, living together with a partner, living in an urban environment, and smoking daily. The mean HSI was highest (3.0) in Greece and lowest (2.1) in England and the Netherlands.

**Table 1:** Distribution of sociodemographic, socioeconomic and smoking-related characteristics by country

	*Germany*	*Greece*	*Hungary*	*Poland*	*Romania*	*Spain*	*England*	*Netherlands*
	*N=1003*	*N=1000*	*N=1000*	*N=1006*	*N=1001*	*N=1001*	*N=3503*	*N=1213*
**Sex** % (n)								
female	39.1 (392)	46.8 (468)	40.9 (409)	44.5 (448)	41.6 (416)	42.7 (427)	45.9 (1607)	50.8 (617)
male	60.9 (611)	53.2 (532)	59.1 (591)	55.5 (558)	58.4 (585)	57.3 (574)	54.1 (1895)	49.2 (596)
frequency missing (n)	(0)	(0)	(0)	(0)	(0)	(0)	(1)	(0)
**Age group** % (n)								
18–24	8.4 (84)	8.4 (84)	9.2 (92)	8.0 (80)	14.3 (143)	12.1 (122)	16.8 (589)	10.8 (131)
25–39	25.6 (256)	28.9 (289)	33.9 (339)	33.5 (337)	38.3 (383)	29.0 (290)	32.3 (1133)	22.8 (277)
40–54	36.5 (366)	35.6 (356)	33.5 (335)	29.5 (297)	30.9 (309)	38.5 (385)	26.2 (919)	27.2 (330)
55+	29.5 (296)	27.2 (272)	23.4 (233)	29.0 (292)	16.6 (166)	20.4 (204)	24.6 (862)	39.2 (475)
frequency missing (n)	(0)	(0)	(0)	(0)	(0)	(0)	(1)	(0)
**Education** % (n)								
low	49.6 (497)	30.2 (301)	64.7 (646)	11.8 (117)	24.8 (245)	44.2 (442)	20.3 (686)	22.9 (273)
moderate	42.4 (425)	49.0 (489)	29.2 (292)	77.5 (767)	63.0 (623)	47.9 (479)	66.0 (2237)	44.9 (535)
high	7.9 (79)	20.8 (208)	6.1 (60)	10.8 (106)	12.2 (121)	7.9 (79)	13.7 (464)	32.2 (384)
frequency missing (n)	(2)	(2)	(2)	(16)	(12)	(1)	(115)	(20)
**Marital status** % (n)								
not married	37.3 (375)	33.6 (336)	33.5 (334)	33.9 (337)	32.5 (325)	41.1 (411)	50.4 (1751)	42.1 (503)
living with partner/ married	62.7 (628)	66.4 (663)	66.6 (664)	66.1 (656)	67.5 (675)	58.9 (590)	49.6 (1720)	57.9 (690)
frequency missing (n)	(0)	(1)	(2)	(13)	(1)	(0)	(32)	(20)
**Level of urbanization** % (n)								
rural	19.4 (195)	22.2 (222)	33.5 (335)	37.2 (374)	37.6 (377)	26.4 (264)	†	†
intermediate	38.7 (388)	51.8 (518)	37.4 (374)	23.0 (231)	19.3 (193)	23.6 (237)	†	†
urban	41.9 (420)	26.0 (260)	29.1 (291)	39.8 (400)	43.1 (431)	50.0 (500)	†	†
frequency missing (n)	(0)	(0)	(0)	(0)	(0)	(0)		
**Smoking status** % (n)								
less than daily	11.7 (117)	3.1 (31)	1.1 (11)	3.6 (37)	5.2 (52)	2.8 (28)	16.7 (586)	8.5 (103)
daily	88.4 (886)	96.9 (969)	98.9 (989)	96.4 (969)	94.8 (949)	97.2 (973)	83.3 (2916)	91.5 (1110)
frequency missing (n)	(0)	(0)	(0)	(0)	(0)	(0)	(1)	(0)
**HSI**^1)^ mean (SD)	2.4 (1.5)	3.0 (1.6)	2.9 (1.3)	2.7 (1.4)	2.9 (1.4)	2.3 (1.6)	2.1 (1.5)	2.1 (1.5)
frequency missing (n)	(121)	(30)	(12)	(68)	(54)	(32)	(386)	(42)

1) HSI: Heaviness of smoking index; ranges from 0 to 6; calculated by summing the value of the categorical cigarettes per day and categorical time to first cigarette, both having category values from 0 to 3. 2) The Netherlandish survey asked for serious quit attempt(s).

† Not captured in survey.

Awareness of tobacco marketing and anti-smoking information in various media and localities, as well as support for tobacco advertising and display bans inside shops and stores by country are presented in Table 2. The percentage of smokers noticing things that promote smoking in the last six months varied widely: it was highest in the Netherlands (69.2 %) and lowest in Hungary (15.4 %) (see also Supplementary Figure 1 in Appendix, for distributions of frequency categories). TAPS were most commonly observed at points of sale, while it was rarely noticed on TV, radio or in print media. Awareness of TAPS was especially high in Germany, where more than a third of smokers noticed TAPS on posters/billboards (38.6 %) as well as outside (34.6 %) or inside (40.3 %) shops that sell tobacco. Awareness of tobacco display inside shops or stores in the last 30 days was highest in Romania (72.3 %), followed by Germany (67.0 %) and Spain (60.9 %), and lowest by a wide margin in England (14.7%).

**Table 2:** Awareness of tobacco marketing and anti-smoking information in various media and localities, and support of tobacco advertising and display bans at points of sale, percentage of all respondents

	*Germany*	*Greece*	*Hungary*	*Poland*	*Romania*	*Spain*	*England*	*Netherlands*
**Noticed things that promote smoking in last 6 months**	53.4	25.8	15.4	34.5	40.8	36.9	41.7	69.2
on TV	8.5	2.3	3.7	10.7	16.1	10.4	†	16.9
on radio	2.4	1.0	2.3	5.5	5.5	4.5	†	2.8
in newspapers or magazines	19.1	3.4	2.6	6.7	8.4	4.4	†	6.8
in social media or on internet	14.5	5.1	3.2	9.4	14.1	6.4	5.2	12.7
on posters or billboards	38.6	9.3	1.7	6.0	13.7	4.7	†	†
in bars or pubs	15.4	4.7	1.4	8.4	11.4	13.1	6.8	†
outside shops or stores that sell tobacco	34.6	15.8	3.4	8.4	16.6	9.9	6.3	†
inside shops or stores that sell tobacco	40.3	16.9	5.3	11.6	18.6	14.8	8.6	†
at events (fairs, markets, festivals, sports, concerts)	10.5	2.0	1.0	4.3	9.4	8.9	†	†
**Noticed display of cigarette or RYO tobacco packages inside shops or stores in last 30 days**	67.0	37.1	29.0	49.9	72.3	60.9	14.7	51.7
**Noticed advertising or information on the dangers of smoking or that encourages quitting in the last 6 months**	45.9	37.3	32.1	48.9	61.5	31.1	†	75.7
**Support complete ban on tobacco advertisements inside shops and stores**	41.5	53.1	63.3	68.0	57.0	32.2	†	45.6
**Support complete ban on display of cigarettes inside shops and stores**	30.0	53.2	56.2	49.4	47.4	30.9	†	42.8

† Question was not asked in survey.

The percentage of smokers noticing advertising or information on the dangers of smoking or that encourages quitting also varied widely (question not asked in England). It was highest in the Netherlands (75.7 %), and lowest in Spain (31.1 %) and Hungary (32.1 %).

Some ITC surveys allowed comparison of noticing anti-smoking information versus noticing things that promote smoking, as a rough measure of ‘net effect’ of anti-smoking versus pro-smoking information, as reported by respondents. Germany and Spain were the only countries where the percentage of smokers noticing anti-smoking information was lower than the percentage of smokers noticing things that promote smoking (Germany 45.9% vs 53.4%; Spain 31.1% vs 36.9%, respectively).

Complete bans on tobacco advertising inside shops and stores, where assessed, were supported by a majority of smokers in Poland (68.0 %), Hungary (63.3 %), Romania (57.0 %), and Greece (53.1 %). In Spain, the support for this type of ban was lowest (32.2 %). Endorsement of cigarette display bans inside shops and stores was overall lower but also above 50 % in Greece and Hungary, whereas in Spain and Germany only 30.9 % and 30.0 %, respectively, endorsed such a ban. Of note, these two countries with the lowest support of a display ban were among the countries with the highest percentage of smokers noticing display of tobacco at points of sale.

Correlates of recalling having noticed things that promote smoking with sociodemographic factors and heaviness of smoking are shown in Table 3. For most of the associations patterns were consistent across countries. In most countries, female smokers tended to notice promotion of smoking less frequently, but statistically significant sex differences were only seen for England, with an adjusted Odds Ratio (aOR) of 0.71 for female versus male smokers, and 95 % confidence intervals (95% CI) ranging from 0.61 to 0.82. In all countries, except the Netherlands, a clear age gradient was observed, with younger smokers being more likely to notice promotion of smoking. A clear educational gradient was only seen in Spain, England and the Netherlands, where lower educated smokers were about 30 to 50 % less likely to notice things that promote smoking. Smokers living in urban areas were more likely to report exposure to things that promote smoking compared to smokers living in rural areas. For HSI, a clear gradient was only seen for Greece, where smokers with low HSI values were twice as likely to notice things that promote smoking (aOR=1.96, 95 % CI: 1.13–3.39).

**Table 3:** Association of having noticed things that promote smoking with sociodemographic factors and heaviness of smoking; percentages and adjusted Odds Ratios from logistic regression models

	*Germany N= 880*		*Greece N= 966*		*Hungary N= 984*		*Poland N= 920*	
	*%*	*aOR (95 % CI)*	*%*	*aOR (95 % CI)*	*%*	*aOR (95 % CI)*	*%*	*aOR (95 % CI)*
**Sex**								
female	49.9	0.78 (0.59–1.04)	25.3	0.89 (0.66–1.20)	15.3	0.94 (0.66–1.36)	33.9	1.04 (0.78–1.39)
male	54.1	1.00	26.2	1.00	15.7	1.00	35.1	1.00
**Age group**								
18–24	61.2	1.69 (0.99–2.91)	36.5	1.98 (1.06–3.69)	18.2	1.91 (0.95–3.84)	51.3	2.57 (1.44–4.58)
25–39	55.1	1.52 (1.05–2.21)	30.0	1.66 (1.06–2.60)	17.8	1.94 (1.16–3.25)	37.6	1.61 (1.12–2.31)
40–54	54.4	1.46 (1.04–2.03)	26.2	1.50 (0.99–2.27)	16.4	1.78 (1.06–3.01)	33.1	1.15 (0.79–1.68)
55+	45.5	1.00	17.5	1.00	10.0	1.00	28.3	1.00
**Education**								
low	52.6	1.06 (0.62–1.81)	18.7	0.89 (0.55–1.43)	14.0	0.72 (0.35–1.47)	23.0	0.94 (0.47–1.85)
moderate	52.4	1.02 (0.59–1.75)	29.8	1.28 (0.87–1.89)	18.3	0.95 (0.46–1.97)	37.2	1.80 (1.08–3.02)
high	52.9	1.00	26.4	1.00	19.2	1.00	27.7	1.00
**Marital status**								
not married	56.0	1.27 (0.95–1.69)	28.9	1.10 (0.79–1.52)	15.6	1.02 (0.69–1.49)	38.1	1.15 (0.85–1.57)
living with partner/married	50.4	1.00	24.2	1.00	15.5	1.00	32.8	1.00
**Level of urbanization**								
rural	58.9	1.20 (0.83–1.74)	22.5	0.66 (0.43–1.03)	13.6	0.87 (0.55–1.38)	26.2	0.44 (0.32–0.62)
intermediate	47.8	0.76 (0.56–1.03)	23.8	0.67 (0.48–0.95)	16.5	1.05 (0.69–1.61)	35.5	0.74 (0.51–1.06)
urban	54.0	1.00	32.4	1.00	16.5	1.00	42.3	1.00
**Heaviness of smoking (HSI)^1)^**								
low (0–1)	55.6	2.52 (1.38–4.63)	34.3	1.96 (1.13–3.39)	20.4	1.13 (0.52–2.48)	29.1	0.91 (0.44–1.91)
moderate (2–4)	53.0	2.12 (1.20–3.74)	25.5	1.42 (0.90–2.23)	14.7	0.80 (0.42–1.53)	36.5	1.46 (0.76–2.82)
high (5–6)	35.0	1.00	18.4	1.00	16.9	1.00	26.1	1.00
**Sex**								
female	38.9	0.91 (0.69–1.20)	35.4	0.89 (0.67–1.17)	35.9	0.71 (0.61–0.82)	70.9	1.06 (0.82–1.37)
male	43.3	1.00	37.5	1.00	44.5	1.00	69.2	1.00
**Age group**								
18–24	48.1	1.80 (1.09–2.97)	49.5	2.09 (1.23–3.52)	55.9	2.54 (1.99–3.26)	72.0	0.97 (0.60–1.56)
25–39	45.2	1.88 (1.26–2.81)	36.8	1.24 (0.83–1.85)	45.1	1.62 (1.33–1.98)	70.3	0.94 (0.67–1.33)
40–54	39.8	1.46 (0.96–2.23)	35.7	1.26 (0.86–1.84)	34.1	1.06 (0.86–1.31)	70.8	1.02 (0.74–1.41)
55+	30.6	1.00	30.5	1.00	33.0	1.00	69.0	1.00
**Education**								
low	38.1	0.99 (0.62–1.59)	28.7	0.50 (0.29–0.84)	36.5	0.71 (0.54–0.92)	60.8	0.58 (0.40–0.83)
moderate	43.2	1.23 (0.80–1.89)	42.7	0.78 (0.47–1.31)	40.5	0.73 (0.58–0.91)	73.0	1.03 (0.75–1.41)
high	39.8	1.00	45.6	1.00	47.9	1.00	72.7	1.00
**Marital status**								
not married	48.0	1.35 (1.00–1.83)	37.7	0.85 (0.63–1.14)	42.1	1.00 (0.86–1.17)	73.6	1.30 (0.98–1.72)
living with partner/married	38.4	1.00	35.8	1.00	39.1	1.00	67.6	1.00
**Level of urbanization**								
rural	38.8	0.72 (0.53–0.97)	26.7	0.68 (0.48–0.96)	†	†	†	†
intermediate	35.7	0.64 (0.45–0.93)	43.4	1.33 (0.96–1.84)	†	†	†	†
urban	46.4	1.00	38.7	1.00	†	†	†	†
**Heaviness of smoking (HSI)^1)^**								
low (0–1)	39.7	0.99 (0.55–1.76)	40.5	0.68 (0.41–1.14)	45.0	0.82 (0.57–1.19)	69.3	0.92 (0.50–1.68)
moderate (2–4)	41.9	1.08 (0.67–1.75)	32.9	0.52 (0.32–0.84)	37.5	0.72 (0.50–1.02)	70.8	1.06 (0.60–1.90)
high (5–6)	41.0	1.00	46.6	1.00	43.9	1.00	67.3	1.00

Percentages are not adjusted for covariates. ORs are adjusted for all covariates listed in the table.

1) HSI: Heaviness of smoking index; ranges from 0–6; calculated by summing the value of the categorical cigarettes per day and categorical time–first cigarette, both having category values from 0–3.

† Not captured in survey.

## DISCUSSION

### Results in context

The analyses showed a wide variety of awareness of both TAPS and anti-smoking information across countries. When comparing country-specific regulations regarding TAPS and through the Tobacco Control Scale^12^ domain ‘bans of tobacco advertising’ (Table 4), TAPS tended to be noticed more often in countries with less restrictive regulation (e.g. Germany and Greece). In Germany, the only country within the EU where outdoor tobacco advertising is still allowed, the percentage of smokers having noticed tobacco advertising on billboards was also markedly higher compared to other media and countries.

**Table 4:** Bans (■) on selected direct and indirect tobacco advertising, promotion and sponsorship in 2016 by country

	*DE*	*GR*	*HU*	*PL*	*RO*	*ES*	*EN*	*NL*
**Bans on direct tobacco marketing**								
National TV and radio	■	■	■	■	■	■	■	■
National newspapers and magazines	■	■	■	■	■	■	■	■
Internet	■	■	■	■	■	■	■	■
Billboards and outdoor advertising	○	■	■	■	■	■	■	■
Ambient media^1)^	○	○	■	■	■	■	■	■
Points of sale	○	○	■	■	■	○	■	○
**Bans on indirect tobacco marketing**								
Promotional activities (e.g. at events)	○	■	■	■	○	■	■	■
Sponsorship	○	○	■	■	○	■	■	■
Display of tobacco products outside POS^2)^	○	○	■	■	○	■	■	○
Display of tobacco products inside POS	○	○	○	○	○	○	■	○
Internet sales of tobacco products	○	■	■	■	○	■	○	○
TCS^3)^ 2016 Advertising Score^12^	4	6	11	11	8	9	12	9

DE: Germany, GR: Greece, HU: Hungary, PL: Poland, RO: Romania, ES: Spain, EN: England, NL: Netherlands | ■: ban existent, ○: no ban

1) Ambient media: out-of-homeproducts that are utilised for advertising – generally in the direct living environment of the target group

2) POS: points of sale

3) TCS: Tobacco Control Scale.

While exposure to individual TAPS channels was also reported in countries with more comprehensive advertising bans (e.g. Hungary and England), this was generally at lower levels compared to countries with less comprehensive bans. These findings are consistent with a previous study using data from the EU-wide 2014 Eurobarometer survey among the general population, which showed that those living in countries with more comprehensive advertising bans were less likely to report exposure to tobacco advertising in the last twelve months^23^. This supports the conclusion that TAPS bans are effective in reducing exposure to marketing activities for tobacco products.

Although tobacco advertising is banned on TV and radio, in print media, and on the internet, in all countries included in this analysis, substantial proportions of the surveyed smokers (up to 19.1 %) have nevertheless noticed advertising in these media. Also, tobacco advertising exposure was quite common outside and inside of points of sale, even in countries where bans on this kind of advertising have been implemented (Hungary, Romania, England). The same applies to the display of tobacco products inside shops and stores in England, which quite a few respondents reported to have noticed even though it is banned in this country. While some misreporting cannot be ruled out due to inaccurate recall or other causes, and some of the exposure could be due to non-TAPS sources that are also captured by asking for ‘things that promote smoking’, the prevalence of self-reported exposure, despite bans being in place, could possibly point to the exploitation of loopholes or to problems with enforcement.

The multivariate analysis revealed some variation of self-reported exposure to tobacco promotion with sociodemographic factors, of which the age pattern showed the largest consistence across countries with younger smokers being more likely to notice tobacco promotion than older smokers. This is in line with the recently published study using data from the EU-wide Eurobarometer Survey, which showed a clear age gradient and noted the highest self-reported TAPS exposure among 15- to 24-yearolds^23^.

It is noteworthy that support for complete bans on tobacco advertising and on display of tobacco products inside points of sale was moderate to high and tended to be higher in countries where advertising bans at the point of sale were in place. It has been found for smoke-free legislation that comprehensive policies attract more support from smokers than partial policies^24^, and it is possible that this applies to advertising bans as well.

### Limitations and strengths

Some limitations need to be considered when interpreting the results of this study. First, this study is based on self-reported recall of exposure to TAPS. This measure can be subject to recall bias and in some cases might reflect awareness to TAPS rather than actual exposure. However, self-reported exposure is widely used as a standard method in surveys on TAPS, which makes our results comparable with other studies.

Second, our TAPS exposure measurement captured ‘things that promote smoking’, which does not necessarily include TAPS alone, but could also include other ways of favourable depiction of smoking, such as through news articles or movies.

Third, the media-specific exposure variable used in this study was based on a simple yes/no question and does not capture frequency of exposure. This needs to be considered when interpreting country differences, as self-reported exposure to TAPS in a country with stronger regulations might reflect a much less frequent actual exposure to TAPS than self-reported exposure in a country with less regulations. The country differences in terms of actual exposure to TAPS might therefore even be larger than found in this study.

Finally, this study is based on cross-sectional samples and thus can only show associations while not allowing any conclusions to be made on the direction of these associations.

On the other hand, the major strength of this study is that the surveys were based on large national probability samples of smokers from eight European countries, using standardized survey questions that assure comparisons across countries.

## CONCLUSIONS

Exposure to tobacco marketing varied widely between countries. Despite the cross-sectional design precluding causal conclusions, the findings indicate a negative association between comprehensiveness of TAPS legislation and exposure to tobacco marketing. However, significant exposure was found even in countries with more comprehensive TAPS legislation, indicating a need for stronger enforcement and closing of loopholes in line with FCTC guidelines^25^. As TAPS has been shown to reinforce smoking, this might help smokers who intend to cut down or quit smoking. Many smokers would even support stronger regulations.

## CONFLICTS OF INTEREST

The authors declare that they have no competing interests, financial or otherwise, related to the current work. K. Przewoźniak reports grants and personal fees from the Polish League Against Cancer, outside the submitted work. C. I. Vardavas reports that he is the Strategic Development Editor of TID and that there are no conflicts of interest with this current work. The rest of the authors have also completed and submitted an ICMJE form for disclosure of potential conflicts of interest.

## References

[1] Saffer H, Chaloupka F (2000). The effect of tobacco advertising bans on tobacco consumption. J Health Econ.

[2] U.S. Department of Health and Human Services (2012). Preventing tobacco use among youth and young adults: a report of the Surgeon General.

[3] Lovato C, Watts A, Stead LF (2011). Impact of tobacco advertising and promotion on increasing adolescent smoking behaviours. Cochrane Database Syst Rev.

[4] Forsyth SR, Kennedy C, Malone RE (2013). The effect of the internet on teen and young adult tobacco use: a literature review. J Pediatr Health Care.

[5] Hebert ET, Vandewater EA, Businelle MS, Harrell MB, Kelder SH, Perry CL (2018). Real Time Assessment of Young Adults’ Attitudes toward Tobacco Messages. Tob Regul Sci.

[6] National Cancer Institute (2008). The role of the media in promoting and reducing tobacco use. Vol 19. Tobacco Control Monograph. No. 07-6242.

[7] Clattenburg EJ, Elf JL, Apelberg BJ (2013). Unplanned cigarette purchases and tobacco point of sale advertising: a potential barrier to smoking cessation. Tob Control.

[8] Siahpush M, Shaikh RA, Smith D (2016). The Association of Exposure to Point-of-Sale Tobacco Marketing with Quit Attempt and Quit Success: Results from a Prospective Study of Smokers in the United States. Int J Environ Res Public Health.

[9] Germain D, McCarthy M, Wakefield M (2010). Smoker sensitivity to retail tobacco displays and quitting: a cohort study. Addiction.

[10] Levy DT, Chaloupka F, Gitchell J (2004). The effects of tobacco control policies on smoking rates: a tobacco control scorecard. J Public Health Manag Pract.

[11] World Health Organization (2003). WHO Framework Convention on Tobacco Control.

[12] Joosens L, Raw M The Tobacco Control Scale 2016 in Europe.

[13] European Parliament, European Council (2003). Directive 2003/33/EC of the European Parliament and of the Council of 26 May 2003 on the approximation of the laws, regulations and administrative provisions of the Member States relating to the advertising and sponsorship of tobacco products.

[14] European Network for Smoking and Tobacco Prevention (ENSP) European Regulatory Science on Tobacco: Policy implementation to reduce lung diseases (EUREST-PLUS).

[15] Vardavas CI, Bécuwe N, Demjén T (2018). Study Protocol of European Regulatory Science on Tobacco (EURESTPLUS): Policy implementation to reduce lung disease. Tobacco Induced Diseases.

[16] European Parliament, European Council (2014). Directive 2014/40/EU of the European Parliament and of the Council of 3 April 2014 on the approximation of the laws, regulations and administrative provisions of the Member States concerning the manufacture, presentation and sale of tobacco and related products and repealing Directive 2001/37/EC.

[17] Fong GT, Thompson ME, Boudreau C (2018). The Conceptual Model and Methods of Wave 1 (2016) of the EUREST-PLUS ITC 6 European Countries Survey. Tobacco Induced Diseases.

[18] Thompson ME, Fong GT, Hammond D (2006). Methods of the International Tobacco Control (ITC) Four Country Survey. Tob Control.

[19] ITC Project (2017). ITC 6 European Country Wave 1 (2016) Technical Report.

[20] ITC Project (2017). ITC Four Country Tobacco and Vaping Survey Wave 1 (2017) Technical Report.

[21] ITC Project (2018). ITC Netherlands Wave 9-11 Technical Report.

[22] Heatherton TF, Kozlowski LT, Frecker RC, Rickert W, Robinson J (1989). Measuring the heaviness of smoking: using self-reported time to the first cigarette of the day and number of cigarettes smoked per day. Br J Addict.

[23] Filippidis FT, Laverty AA, Fernández E, Mons U, Tigova O, Vardavas CI (2017). Correlates of self-reported exposure to advertising of tobacco products and electronic cigarettes across 28 European Union member states. Tob Control.

[24] Mons U, Nagelhout GE, Guignard R (2012). Comprehensive smoke-free policies attract more support from smokers in Europe than partial policies. Eur J Public Health.

[25] World Health Organization (2008). Guidelines for implementation of Article 13: Tobacco advertising, promotion and sponsorship WHO Framework Convention on Tobacco Control.

