# Supplementary material for: Extent and correlates of self-reported exposure to tobacco advertising, promotion and sponsorship in smokers: Findings from the EUREST-PLUS ITC Europe Surveys
Source: Tob Induc Dis. 2019 Jan 17;16:A7. doi: 10.18332/tid/94828 (PMC6519076; doi:10.18332/tid/94828)
Supplement: Supplementary file 1 [file TID-16-A7-s1.pdf]

Supplemental Figure 1. Frequency of having noticed things that promote smoking in the last six months by country

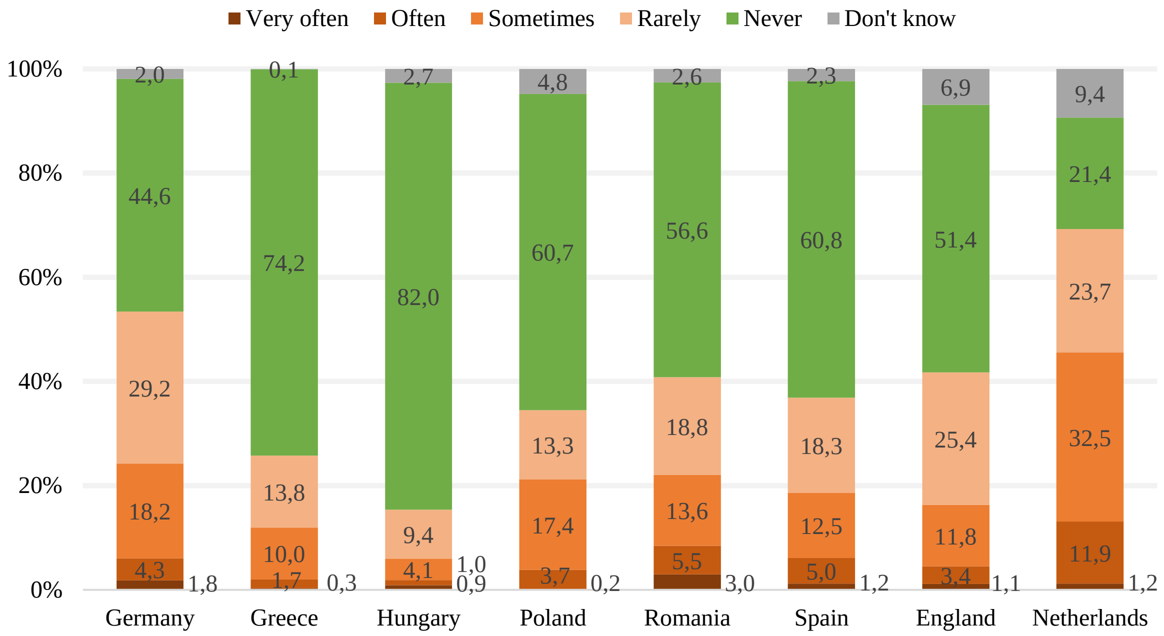

© 2019 Kahnert S.
